# Long-term survival following transvenous lead extraction: Importance of indication and comorbidities

**DOI:** 10.1016/j.hrthm.2021.05.007

**Published:** 2021-05-10

**Authors:** Vishal S. Mehta, Mark K. Elliott, Baldeep S. Sidhu, Justin Gould, Tiffany Kemp, Vittoria Vergani, Suraj Kadiwar, Anoop Kumar Shetty, Christopher Blauth, Jaswinder Gill, Paolo Bosco, Christopher A. Rinaldi

**Affiliations:** * Cardiology Department, Guy’s and St Thomas’ NHS Foundation Trust, London, United Kingdom; † School of Biomedical Engineering and Imaging Sciences, King’s College London, United Kingdom

**Keywords:** Infection, Mortality, Prognosis, Transvenous lead extraction

## Abstract

**Background:**

Long-term outcomes are poorly understood, and data in patients undergoing transvenous lead extraction (TLE) are lacking.

**Objective:**

The purpose of this study was to evaluate factors influencing survival in patients undergoing TLE depending on extraction indication.

**Methods:**

Clinical data from consecutive patients undergoing TLE in the reference center between 2000 and 2019 were prospectively collected. The total cohort was divided into groups depending on whether there was an infective or noninfective indication for TLE. We evaluated the association of demographic, clinical, and device-related and procedure-related factors on mortality.

**Results:**

A total of 1151 patients were included. Mean follow-up was 66 months, and mortality was 34.2% (n = 392). Of these patients, 632 (54.9%) and 519 (45.1%) were for infective and noninfective indications, respectively. A higher proportion in the infection group died (38.6% vs 28.5%; *P* <.001). In the total cohort, multivariable analysis demonstrated increased mortality risk with age >75 years (hazard ratio [HR] 2.98; 95% confidence interval [CI] 2.35–3.78; *P* <.001), estimated glomerular filtration rate <60 mL/min/1.73 m^2^ (HR 1.67; 95% CI 1.31–2.13; *P* <.001), higher cumulative comorbidity (HR 1.17; 95% CI 1.09–1.26; *P* <.001), reduced risk per percentage increase in left ventricular ejection fraction (HR 0.98; 95% CI 0.97–0.99; *P* <.001), and near unity per year of additional lead dwell time (HR 0.98; 95% CI 0.96–1.00; *P* = .037). Kaplan-Meier survival curves demonstrated worse prognosis, with a higher number of leads extracted and increasing comorbidities.

**Conclusion:**

Long-term mortality for patients undergoing TLE remains high. Consensus guidelines recommend evaluating risk for major complications when determining whether to proceed with TLE. This study suggests also assessing longer-term outcomes when considering TLE in those with a high risk of medium- and long-term mortality, particularly for noninfective indications.

## Introduction

The rise in the use of cardiac implantable electronic devices (CIEDs) has been paralleled by an increase in the number of procedures required for the removal of such devices and their associated leads.^1^ Transvenous lead extraction (TLE) forms the basis of the management of infected CIEDs, and malfunctioning and redundant leads.^2^ High procedural success rates with low rates of major in-hospital complications as achieved in ELECTRa (European Lead Extraction ConTRolled Registry) demonstrate complete clinical success of 96.7% and an in-hospital major complication rate of 1.7%.^3^ Overall hospital mortality was low at 1.4%, with procedure-related mortality of 0.5%. Longer-term mortality following transvenous lead extraction is less well described, and few registry analyses have assessed the incidence of, and factors determining, long-term mortality post TLE.^4,5^

Longer-term outcomes are important, as they should inform the decision-making and consent process, especially in noninfected cases for which there may not be a class I indication for lead removal. We set out to assess long-term mortality following TLE and predictors of mortality in relation to underlying etiology. We studied data from a single, high-volume tertiary referral center for TLE with regard to long-term mortality and potential correlates.

## Methods

All consecutive patients undergoing TLE who survived to discharge in a high-volume center in the United Kingdom were prospectively recorded onto a computer database between October 2000 and November 2019. Multiple parameters were recorded, including demographics, extraction indication, device and lead type, comorbidities (CMs), biochemistry and pathology results, procedural success, major complications, and technical extraction information. Mortality was recorded retrospectively by linking unique patient registration numbers (National Health Service [NHS] numbers) and the Office for National Statistics (ONS) mortality data updated as of February 2020. ONS mortality data are considered the gold standard for mortality records in the United Kingdom.^6^ The database collection and analysis were approved by the Institutional Review Board of Guy’s and St Thomas’ Hospital. The current analysis is split according to TLE indication as total cohort—any indication; infection group—infective indication; or noninfection group—noninfective indication

### Definitions

TLE was defined per the EHRA (European Heart Rhythm Association) and Heart Rhythm Society guidelines.^7^ The 2018 EHRA guidelines defined the extraction indication, procedural success, and complication rate.^2^ The extraction procedure undertaken at this center has been described in detail elsewhere.^8^ If there was more than 1 indication for lead extraction or original implantation indication, this was counted independently. Number of previous device interventions was defined as the number of CIED procedures undertaken on the patient before the recorded lead extraction. Each patient was only included once, based on the latest TLE procedure date. Lead dwell time was calculated as the oldest targeted lead *in situ* at the time of extraction. Follow-up time and age were calculated from date of TLE. Major cardiovascular CMs were recorded. Glomerular filtration rate was estimated by the 4-variable Modification of Diet in Renal Disease equation.^9^

### Statistical analysis

Categorical variables were compared with the χ^2^ test or Fisher exact test. Continuous variables were assessed for normality using an appropriate test. Normally distributed data were analyzed using an independent samples Student *t* test. Non-normally distributed continuous data were analyzed using the Kruskal-Wallis 1-way analysis of variance test. Results are presented as mean ± SD for normally distributed variables and median (interquartile range) for non-normally distributed variables. Categorical variables are presented as number of patients (% of group). Univariable and multivariable cox (proportional hazard) regression was performed to determine predictors of mortality. Results are presented as hazard ratio (HR) (95% confidence interval [CI]; *P* value). Only factors that met the proportional hazards and linear relations assumption as appropriate were included in the final multivariable analysis. Relevant variables found to be statistically significant at univariable analysis alongside covariates considered clinically important were used in the multivariable analysis. A concordance index evaluated the predictions made by the multivariable model and significance determined using the Wald test. Kaplan-Meier survival curves were formulated to estimate unadjusted survival distributions from death and tested with the log-rank test. Across all statistical tests, 2-tailed *P* ≤.05 was considered significant, and CIs were set at 95%. Analyses were performed using R Version 1.3.1093 (The R Foundation).

## Results

### Demographics

A total of 1151 consecutive patients were included. Mean age at explant was 65 ± 14.7 years, and males predominated (72.5%) (Table 1). The most common indication for TLE was infection (n = 632 [53.1%]: 36.8% local and 18.2% systemic infection). Median lead dwell time was 62.9 (20–119) months, with a total of 2375 leads extracted. The mode number of leads extracted per procedure was 2 (n = 505; [43.9%]). The most common indication for original device implantation was bradycardia (n = 560 [48.7%]). Mean left ventricular ejection fraction (LVEF) was 45.4 ± 14. A total of 2190 CMs were recorded (mean 1.9 CMs per patient). The most common CM was hypertension (n = 434 [39.4%]). TLE procedure-related major and minor complication rates were 1.9% and 8.6%, respectively, with a clinical failure rate of 1.0%.

### Mortality at follow-up

During long-term follow-up (mean 66.4 ± 49.9 months), 392 patients (34.1%) died. Kaplan-Meier survival analysis demonstrated a survival probability of 95.7% at 6 months, 93% at 1 year, 87.9% at 2 years, 73.4% at 5 years, and 51.5% at 10 years (Supplemental Figure S1). Patients who died were older (72.4 ± 11.2 years vs 60.9 ± 14.8 years; *P* <.001) and more likely male (79.6% [n = 312] vs 68.8% [n = 522]; *P* <.001). Among patients who died, median lead dwell time was shorter (54 [16–97] vs 71 [23–128] months; *P* <.001). Any infective indication was significant for mortality (*P* <.001), as was local infection (*P* = .004). Increasing burden of leads extracted (*P* = .033), extraction of a left ventricular (LV) lead (*P* <.001), increasing CM burden (*P* <.001), reduced LVEF (40.2 ± 14.3 vs 48.1 ± 13.1; *P* <.001), and higher median creatinine (105 [86–138] vs 86 [72–104] mg/dL; *P* <.001) were all associated with long-term mortality. Clinical failure, partial removal, or complication incidence were not associated with long-term mortality.

### Subgroup analysis: Infectious vs noninfectious indication

Patients undergoing TLE for an infective indication were more likely to be male (77.1% vs 66.9%; *P* <.001), older at explant (67.6 ± 13.6 vs 61.5 ± 15.4 years; *P* <.001), and had more leads extracted than those for noninfectious indications (mean 2.28 vs 1.8; *P* <.001) (Supplemental Table S1). Similar mean CM burdens were observed in both groups (2 vs 1.78; *P* = .478); however, chronic kidney disease (CKD) was more prevalent in the infection group (21.1% vs 15.6%; *P* = .022). Mean LVEF was higher in the infection group (46.4 ± 13.8 vs 44.0 ± 14.6; *P* = .007), as were me-dian creatinine levels (96 [79–121] vs 87 [72–111] mg/dL; *P* <.001). The need for temporary pacing was more prevalent in the infection group (31.6% vs 13.1%; *P* <.001). At follow-up, a higher proportion of patients in the TLE infection vs the non-infection group died (38.6% vs 28.5%; *P* = .004), with survival probability of 90.6% vs 95.9% at 1 year; 84.6% vs 91.8% at 2 years; 70.1% vs 77.4% at 5 years, and 47.6% vs 57.6% at 10 years (*P* = .003) (Supplemental Figure S2). Notably, there was no significant difference in long-term mortality between systemic (*P* = .58) and local infection (*P* = .61).

### Univariable analysis of long-term survival

**Figure 1:**
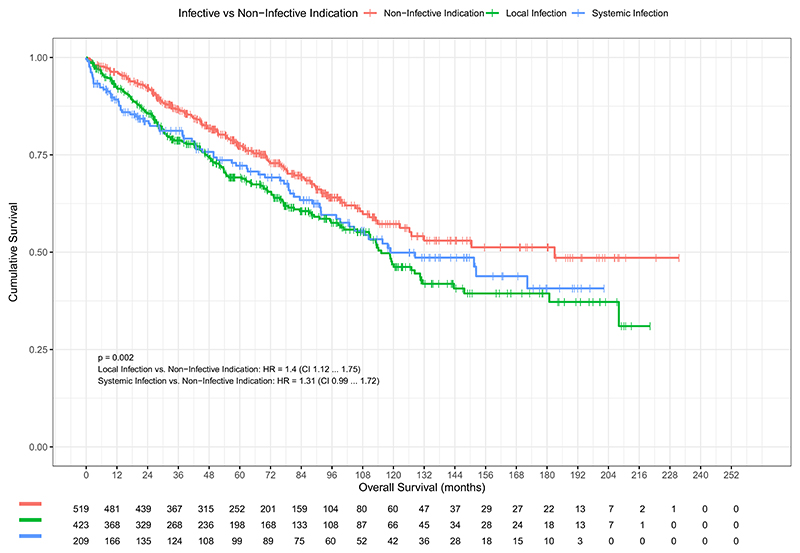
Kaplan-Meier survival probability in patients depending on indication for transvenous lead extraction, with embedded risk table. CI = confidence interval; HR = hazard ratio.

On univariable Cox regression analysis, older age at explant, male gender, shorter lead dwell time, increasing burden of leads, LV leads extracted, lower LVEF, any infective indication, all CMs, increasing burden of CMs, and higher creatinine and C-reactive protein (CRP) all correlated with mortality in the total cohort (Table 2). Any infective indication conferred a significant mortality risk (HR 1.4; 95% CI 1.1–1.7; *P* = .003). Similar HRs depending on indication were observed (local infection vs noninfective: HR 1.3; 95% CI 1.12–1.75, *P* = .003, vs HR 1.4; 95% CI 1.12–1.75, *P* = .058). The difference was primarily accounted for in the first year of follow-up (Figure 1).

**Figure 2:**
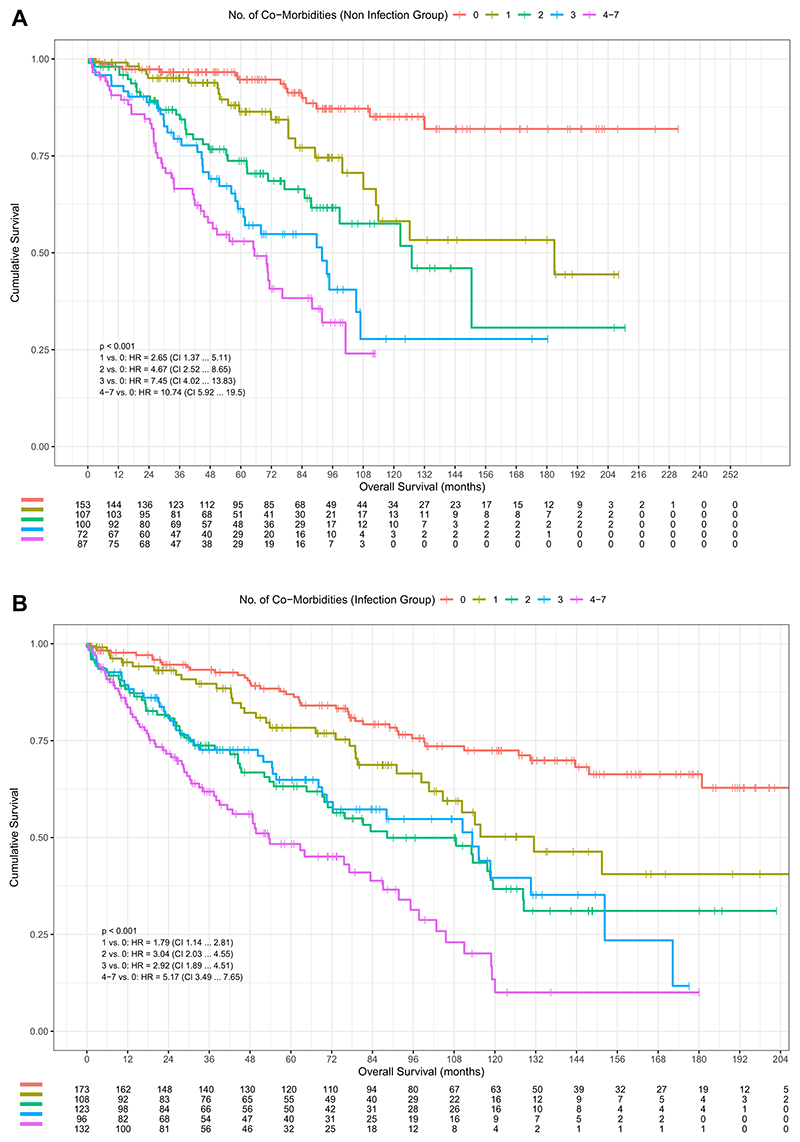
Kaplan-Meier survival probability in patients depending on the total number of comorbidities, with associated risk table. Noninfection group **(A)** and infection group **(B)**. CI = confidence interval; HR = hazard ratio.

**Figure 3:**
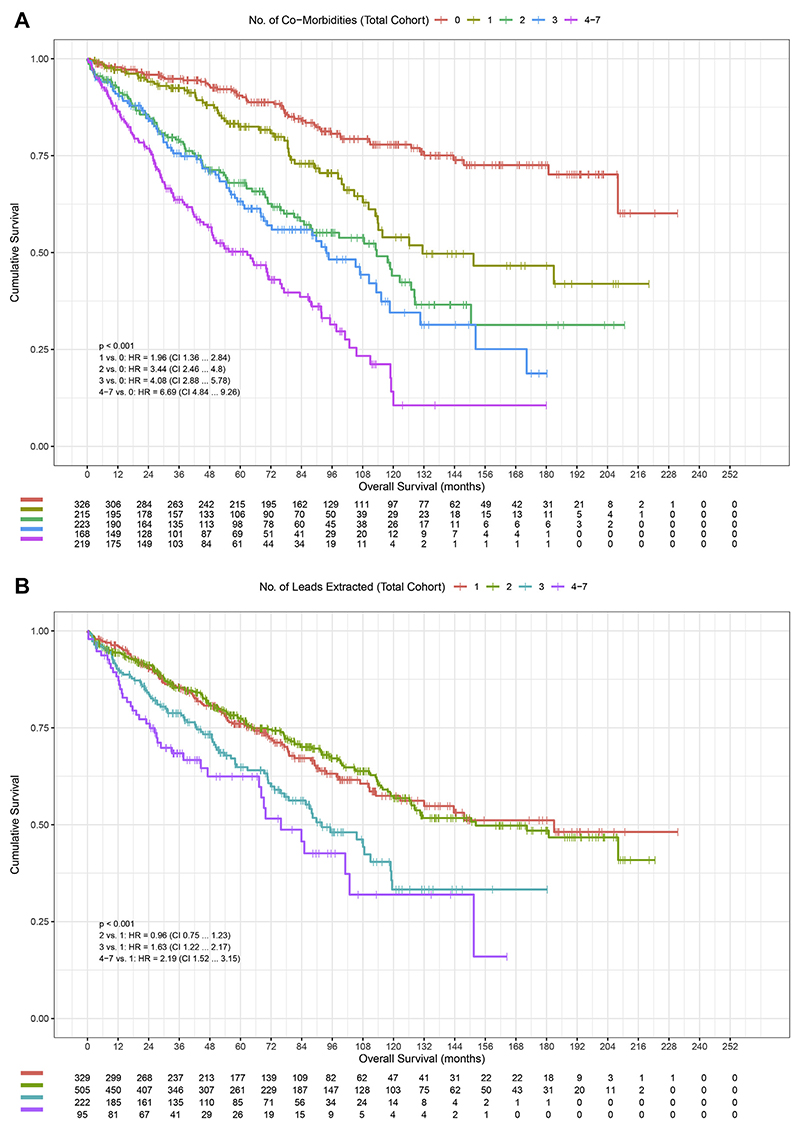
Kaplan-Meier survival probability in the total cohort, with associated risk table. Number of comorbidities **(A)** and number of leads extracted **(B)**. CI = confidence interval; HR = hazard ratio.

**Figure 4:**
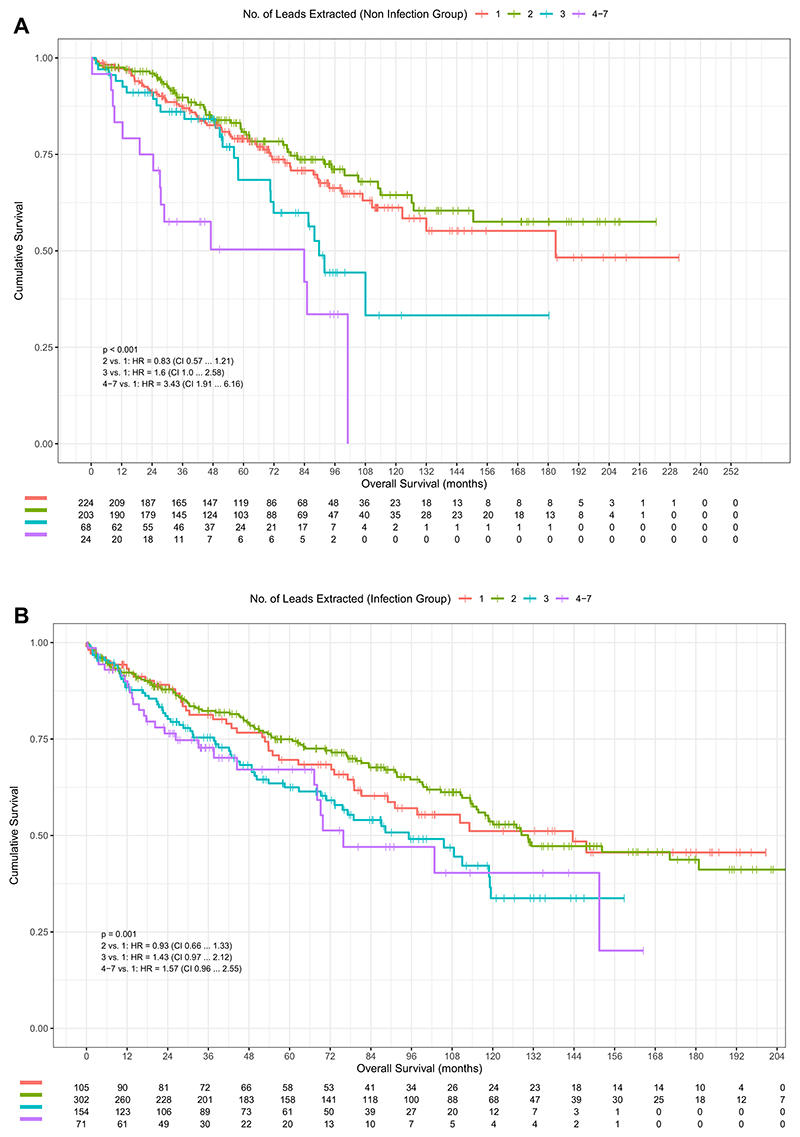
Kaplan-Meier survival probability in patients depending on the total number of leads extracted, with associated risk table. Noninfection group **(A)** and infection group **(B)**. CI = confidence interval; HR = hazard ratio.

The impact of increasing burden of CMs was more pronounced in the noninfection vs infection group (1 vs 0 CM: HR 1.79 vs 2.65; 4–7 vs 0 CM: HR 5.17 vs 10.74; *P* <.001) (Figure 2). The noninfection group compared less favorably than the infection group compared to the total 186 cohort (1 vs 0 CM: HR 1.96; 4–7 vs 0 CM: HR = 6.69; *P* <.001) (Figure 3). In the infection group, the highest risk was associated with CKD (HR 2.9; 95% CI 2.2–3.9; *P* <.001). In the noninfection group, the highest risk was associated peripheral vascular disease (HR 4.1; 95% CI 2.2–7.7; *P* <.001), followed by CKD (HR 3.5; 95% CI 2.4–5.1; *P* <.001) (Supplemental Table S2). The burden of number of leads extracted on mortality was more pronounced in the noninfection group (*P* <.001) (Figure 4), with pairwise HRs of 4–7 leads of HR 1.57; 95% CI 0.96–2.55 (*P* = .072) in infection group vs HR 3.43; 95% CI 1.91–6.16 (*P* <.001) in non-infection group.

### Multivariable analysis of long-term survival

**Figure 5:**
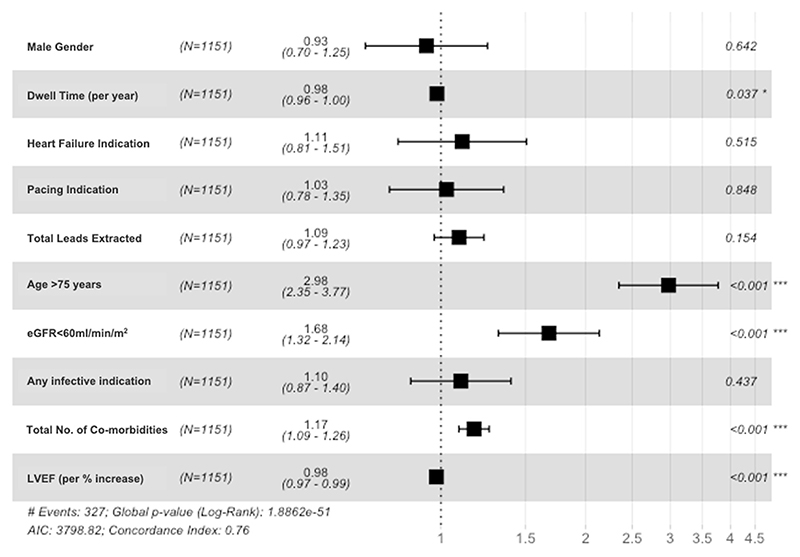
Multivariable cox proportional hazards regression model (*P* <.001) to predict mortality after transvenous lead extraction (TLE) in the total cohort. LVEF = left ventricular ejection fraction. * *P* <.05, *** *P* <.001.

Factors considered clinically important and those close to and reaching statistical significance were included in the multivariable cox regression model to predict mortality after TLE (Table 2). For the total cohort, age >75 years (HR 2.98; 95% CI 2.35–3.78; *P* <.001) (per each additional year: HR 1.05; 95% CI 1.04–1.07; *P* <.001), LVEF per percentage increase (HR 0.98; 95% CI 0.97–0.99; *P* <.001), estimated glomerular filtration rate <60 mL/min/1.73 m^2^ (HR 1.67; 95% CI 1.31–2.13; *P* <.001), shorter lead dwell time (HR 0.98; 95% CI 0.96–1.00; *P* = .034), and higher total CM burden (HR 1.17; 95% CI 1.09–1.85; *P* <.001) were all significant factors predicting mortality (Figure 5). In multivariable analysis of the infection group, higher CRP per mg/L increase at time of TLE (HR 1.01; 95% CI 1.00–1.01; *P* <.001) predicted mortality. Higher total CM burden predicted mortality in the infection and noninfection groups (HR 1.17; 95% CI 1.06–1.28, *P* = .001, vs HR 1.20; 95% CI 1.06–1.37, *P* = .005) (Supplemental Table S3).

## Discussion

An understanding of mortality at follow-up post-TLE is important to evaluate the longer-term implications of the procedure. This analysis is the largest registry study to date comparing long-term mortality of patients undergoing TLE for both infective and noninfective indications.

The main findings are as follows. (1) At 1- year follow-up post-TLE, 93% of patients survived following discharge. However, an infective indication conferred negative overall survival compared with noninfective indications (90.6% vs 95.9%). (2) Multivariable analysis identified commonalities in factors affecting long-term mortality, including higher age at explant, lower LVEF, higher creatinine level, and higher total CM burden. (3) Cumulative burden of CMs and number of leads extracted both were important factors determining long-term survival on univariable analysis; however, lead burden was not significant on multivariable analysis.

### Comparison with previous studies

Most studies of TLE have focused on in-hospital mortality following TLE, with low rates of major complication- and procedure-related mortality. ELECTRa demonstrated similar outcomes for procedural outcomes when compared with this cohort with regard to procedure-related major complications (1.7% vs 1.9%) and failure of TLE procedure (1.5% vs 1.0%).^3^ Longer-term outcomes following lead extraction are less well described. The current study is the largest to look at long-term mortality following TLE and to compare infective vs noninfective indications. CKD was identified as a significant predictor of long-term mortality following TLE. This has been identified by Deharo et al^10^ as a significant risk factor for long-term mortality in patients undergoing TLE (HR 3.31, 95% CI 1.73–3.36), whereas Shah et al^11^ identified end-stage renal disease as a greater long-term risk than renal insufficiency. Multiple studies have demonstrated the independent influence of individual CMs, particularly diabetes mellitus, valvular disease, ischemic heart disease, and CKD; however, few have commented on the burden of cumulative CMs in depth. Habib et al^12^ analyzed mortality based on the Charlson CM score in 415 patients; however, increasing CM burden based on this score did not relate to worsening mortality.

A large retrospective group study of patients undergoing TLE for infective causes by Polewczyk et al^4^ demonstrated HR similar to that in our study with respect to CKD and type 2 diabetes mellitus. Their study also identified the presence of a vegetation that demonstrated no significant higher mortality risk (HR 1.41; 95% CI 0.98–2.05; *P* = .67), in line with the current study (HR 0.82; 95% CI 0.54–1.3; *P* = .37). This could be due to survivor treatment selection bias or more aggressive treatment in those with vegetations identified on imaging. The current study has identified novel predictors of mortality including CM and lead burden.

### Importance of CMs

Across the total cohort, the cumulative burden of CMs was noted to be significantly associated with long-term mortality. Higher creatinine, age at explant, total number of CMs, and lower LVEF were all predictive of mortality. The increasing risk by year of age at explant was particularly marked, with a 4%–5% increased risk of mortality with each increased year at explant. Increasing burden of CMs conferred an even greater mortality risk of 11% or 16% increase per CM in the infection and noninfection group, respectively. Notably, the impact of additional CMs was more pronounced in the noninfection compared with the infection group, with earlier and more noticeable separation of survival curves in the noninfection group compared with the infection group (Figure 2).

### Lead-related data

The burden of number of leads extracted was significant across all groups but was more noticeable in the noninfection group on univariable analysis, as demonstrated by the pairwise HRs (Figure 3). Early curve separation indicates the importance of this when assessing both medium- and long-term mortality in evaluating risk of TLE. Our study differs from the analysis of Maytin et al^13^ of a mixed group of patients, demonstrating no significance associated with burden of lead removal (HR 0.94; 95% CI 0.77–1.14), whereas an assessment by Merchant et al^14^ assessing defibrillator lead extraction did demonstrate significances in a primarily noninfectious population (HR 1.584; 95% CI 1.144–2.192).

Notably, the burden of leads extracted was particularly hazardous on univariable analysis; however, this was minimized on multivariable analysis. This suggests that increased lead burden is less relevant when adjusted for presence of cardiac resynchronization therapy and/or the need for previous device upgrade through LV lead inclusion and presence of heart failure. On univariable analysis, mortality risk is noted to be significantly higher based on whether an LV lead is explanted. This may be due to a negative reverse remodeling effect in these patients who are without resynchronization therapy for a period of time post-TLE. Notably, longer lead dwell time was close to unity on multivariable analysis (HR 0.98; 95T% CI 0.96–1.00; *P* = .037). This is contrary to established short-term outcomes whereby longer lead dwell time is associated with increased procedure-related death.^15^ In our cohort, this is likely due to younger patients with fewer CMs having longer lead dwell times (Supplemental Figure S3).

### Assessment of the infection group

Extraction for an infective cause is well established as representing higher risk of major complication and short-term mortality.^8^ Development of a cardiac device infection has been identified as a major factor in long-term mortality.^16^ Two large-scale observational studies have evaluated factors affecting long-term mortality in CIED infections.^4,12^ These studies demonstrated findings similar to ours in that raised CRP was associated with increased mortality. In our study, local infection was associated with a long-term mortality risk similar to that for systemic infection, which suggests that local infection should be treated as aggressively as systemic infection. Our Kaplan-Meier assessment does show similarities to previous studies,^4,17^ where there is a significant difference in shorter-term mortality as demonstrated by early curve separation.

### Technical aspects of the procedure

As previously described, procedural success of TLE is high and was similarly high in this study, with only a 1.2% clinical failure rate. In a similar manner, both major and minor complication rates related favorably and is the likely reason for their nonsignificant impact on long-term mortality. Use of manual traction only (*P* = .43), and nonpowered compared to powered tool use (*P =*.60) demonstrated no significant difference in mortality. Apart from pacing in the infection group, we were unable to demonstrate a significant difference in long-term mortality across both groups with regard to extraction tool and approach adopted in those patients surviving to discharge.

### Study limitations

The findings of our study are limited by the inherent issues identified with observational studies, namely, the possibility of unidentified confounders. Predictors of long-term mortality for the group were discussed; however, the cause-and-effect relationship remains associative. We opted to only include patients who survived to discharge, which may have introduced survival bias. Additionally, given our cohort size, there was limited power to detect small differences in mortality. Notably, only 20 patients (1.7%) did not survive to discharge. To mitigate this, a model taking into account the competing risk of death was also performed, with no significant difference in the results to the current analysis (Supplemental Figure S4). As our institution is a tertiary care center, referral bias could have affected the clinical data, thereby limiting the generalization of these findings to other patient populations. In the infective group, duration of antibiotic therapy and time since diagnosis of CIED infection would have allowed adjustment for these factors. Causes of death in these patients are unknown.

## Conclusion

Consensus guidelines recommend the evaluation of risk for major complications when determining whether to proceed with TLE. The current study suggests an evaluation of mortality risk, especially for patients with non–class I noninfective indications, should be considered when evaluating the benefit of TLE.^2^ This study demonstrates the need to consider the impact of TLE in patients who have a high risk of medium- and long-term mortality, particularly patients with high lead and CM burdens. These factors should be considered carefully when discussing the risks vs benefits of lead extraction with the multidisciplinary team and the patient.

## Supplementary Material

Supplementary material

Appendix

## Figures and Tables

**Table 1:** Baseline characteristics of the total cohort

	Total cohort			
	Total	Alive	Dead	*P* value
Total no. of patients	1151	759	392	
Follow-up time (mo)	62.90 (20.20–118.80)	70.75 (22.92–127.67)	53.60 (15.50–97.40)	<.001
Male	834 (72.5)	522 (68.8)	312 (79.6)	<.001
Explant age (y)	64.83 ± 14.72	60.94 ± 14.82	72.38 ± 11.19	<.001
Age >75 y	328 (28.5)	136 (17.9)	192 (49.0)	<.001
Lead dwell time (mo)	62.90 (20.20–118.80)	70.75 (22.92–127.67)	53.60 (15.50–97.40)	<.001
Indication for extraction				
Any infective indication	632 (54.9)	388 (51.1)	244 (62.2)	<.001
Local infection	423 (36.8)	256 (33.8)	167 (42.6)	.004
Systemic infection	209 (18.2)	132 (17.4)	77 (19.6)	.396
Noninfective indication				
Lead dysfunction	349 (30.3)	244 (32.1)	105 (26.8)	.071
Functional lead	31 (2.7)	24 (3.2)	7 (1.8)	.236
Lead complication	78 (6.8)	50 (6.6)	28 (7.1)	.817
Lead access	49 (4.3)	34 (4.5)	15 (3.8)	.698
Lead pain	15 (1.3)	14 (1.9)	1 (0.3)	.047
Other indication	105 (9.1)	72 (9.5)	33 (8.4)	.625
Lead type				
Single-coil defibrillator lead	233 (20.2)	150 (19.8)	83 (21.2)	.201
Dual-coil defibrillator lead	239 (28.8)	152 (20.0)	87 (22.2)	.455
No. of LV leads				<.001
1	225 (19.5)	118 (15.5)	107 (27.3)	
2–3	11 (9.5)	9 (1.2)	2 (0.5)	
Total leads extracted*				.092
1	329 (28.6)	226 (29.8)	103 (26.3)	
2	505 (43.9)	345 (45.5)	160 (40.8)	
3	222 (19.3)	134 (17.7)	88 (22.4)	
4-7	95 (8.3)	54 (7.2)	41 (10.5)	
Indication for CIED				
Primary prevention	113 (9.8)	84 (11.1)	29 (7.4)	.06
Secondary prevention	233 (20.2)	168 (22.1)	65 (16.6)	.032
Any pacing indication	560 (48.7)	355 (46.8)	171 (43.6)	.34
Any HF indication	268 (23.3)	142 (18.7)	126 (32.1)	<.001
Echocardiographic findings				
LVEF	45.37 ± 14.02	48.06 ± 13.11	40.20 ± 14.30	<.001
Comorbidities				
Ischemic heart disease	425 (38.3)	223 (30.3)	202 (53.7)	<.001
CABG	143 (12.9)	65 (8.8)	78 (20.9)	<.001
Valve disease	111 (10.0)	58 (7.9)	53 (14.1)	.002
Heart failure	418 (37.6)	226 (30.7)	192 (51.1)	<.001
Diabetes mellitus	174 (15.8)	105 (14.3)	69 (18.7)	.072
Hypertension	434 (39.4)	259 (35.3)	175 (47.6)	<.001
Peripheral vascular disease	43 (3.9)	19 (2.6)	24 (6.5)	.003
Stroke	87 (7.9)	49 (6.7)	38 (10.3)	.048
Chronic respiratory disease	147 (13.3)	89 (12.1)	58 (15.7)	.124
Chronic kidney disease	208 (18.6)	94 (12.7)	114 (30.1)	<.001
Total no. of comorbidities *				<.001
0	326 (28.3)	268 (35.3)	58 (14.8)	
1	215 (18.7)	159 (20.9)	56 (14.3)	
2	223 (19.4)	136 (17.9)	87 (22.2)	
3	168 (14.6)	94 (12.4)	74 (18.9)	
4–7	219 (19.0)	102 (13.5)	117 (29.8)	
Pre-extraction biochemistry				
Creatinine level	92.00 (76.00–117.00)	86.00 (72.00–104.00)	105.00 (86.00–138.25)	<.001
eGFR	67.33 ± 21.26	72.41 ± 18.70	57.49 ± 22.45	<.001
Peak CRP	6.00 (2.00–17.00)	5.00 (1.00–14.00)	8.00 (4.25–20.75)	.001
No. of previous device interventions				.083
1	352 (30.6)	236 (31.1)	116 (29.6)	
2	170 (14.8)	112 (14.8)	58 (14.8)	
>2	154 (13.4)	121 (15.9)	34 (8.7)	
History of previous extraction	128 (11.1)	87 (11.5)	41 (10.5)	.679
Extraction tools*				
Manual traction only	319 (27.7)	218 (28.7)	101 (25.8)	.321
Nonpowered only	206 (17.9)	116 (15.3)	90 (23.0)	.002
Powered only	119 (10.3)	75 (9.9)	44 (11.2)	.544
Powered and nonpowered	507 (44.0)	350 (46.1)	157 (40.1)	.057
Extraction approach				
Inferior approach	117 (10.2)	92 (12.2)	25 (6.4)	.003
Primary femoral approach	14 (1.2)	10 (1.3)	4 (1.0)	.872
Secondary femoral approach	109 (9.5)	88 (11.7)	21 (5.4)	.001
Pacing during extraction				
Temporary pacing wire	268 (23.3)	176 (23.2)	92 (23.5)	.973
Procedural success*				
Complete remove	1024 (89.0)	677 (89.2)	347 (88.5)	.804
Partial removal	115 (10.0)	73 (9.6)	42 (10.7)	.628
Clinical failure	12 (1.0)	9 (1.2)	3 (0.8)	.719
Complications				
All minor complications	99 (8.6)	70 (9.2)	29 (7.4)	.35
Total major complications	22 (1.9)	18 (2.4)	4 (1.0)	.174

Values are given as median (interquartile range), n (%) or mean ± SD unless otherwise indicated.

*P* value comparing alive vs dead groups.

CABG = coronary artery bypass graft; CIED = cardiac implantable electronic device; CRP = C-reactive protein; eGFR = estimated glomerular filtration rate; HF = heart failure; LV = left ventricle; LVEF = left ventricular ejection fraction.

* These categories are mutually exclusive.

**Table 2:** Univariable Cox regression model to predict long-term mortality after TLE in total cohort

	Total cohort	
	HR (CI)	*P* value
Explant age (y) (per year)	1.1 (1.1–1.1)	<.001
Explant age >75 y	3.7 (2.8–4.9)	<.002
Male gender	1.6 (1.2–2)	<.001
Dwell time (y) (per additional year)	0.97 (0.96–0.99)	<.001
Lead type		
Dual-coil defibrillator leads (vs single-coil)	1.1 (0.86–1.5)	.37
No. of LV leads (per additional LV lead)	1.8 (1.5–2.2)	<.001
Total leads extracted (per additional lead)	1.3 (1.1–1.4)	<.001
Indication for CIED		
Primary prevention (vs secondary prevention)	0.99 (0.67–1.5)	.94
Any pacing indication	0.75 (0.61–0.91)	.0042
Any HF indication	2.3 (1.8–2.8)	<.001
Echocardiographic findings		
LVEF (per % increase)	0.97 (0.96–0.98)	<.001
Indication for extraction		
Any infective indication	1.4 (1.1–1.7)	.0025
Local infection (vs no infection)	1.4 (1.12–1.75)	.003
Systemic infection (vs no infection)	1.3 (0.99–1.72)	.058
Comorbidities		
Ischemic heart disease	2.2 (1.8–2.7)	<.001
CABG	1.9 (1.5–2.4)	<.001
Valve disease	1.9 (1.4–2.5)	<.001
Heart failure	2.7 (2.2–3.3)	<.001
Diabetes mellitus	1.7 (1.3–2.2)	<.001
Hypertension	1.8 (1.5–2.2)	<.001
Peripheral vascular disease	2.3 (1.5–3.5)	<.001
Stroke	1.9 (1.4–2.7)	<.001
Chronic respiratory disease	1.7 (1.3–2.2)	<.001
Chronic kidney disease	3.2 (2.5–4)	<.001
Total no. of comorbidities (per comorbidity)	1.4 (1.3–1.5)	<.001
Pre-extraction biochemistry		
Creatinine level (per 10 mg/dL increase)	1 .09 (1.07–1.10)	<.001
eGFR (per unit increase in mL/min/1.73 m^2^)	0.98 (0.97–0.98)	<.001
eGFR <60 mL/min/1.73 m^2^	3.1 (2.5–3.8)	<.001
Peak CRP (per unit increase in mg/L)	1.00 (1.00–1.01)	<.001
Extraction technique		
Manual traction only	1.1 (0.88–1.3)	.43
Nonpowered only (vs powered only)	1.1 (0.77–1.6)	.6
Powered and nonpowered (vs manual traction only)	0.87 (0.67–1.1)	.29
Inferior approach (vs superior approach)	0.92 (0.61–1.4)	.7
Secondary femoral approach (vs primary femoral approach)	0.9 (0.3–2.7)	.85
Temporary pacing wire	1.1 (0.89–1.4)	.34
Procedural success		
Complete remove	0.95 (0.7–1.3)	.76
Partial removal (vs complete removal)	1.1 (0.78–1.5)	.68
Clinical failure	0.52 (0.17–1.6)	.25
All minor complications (vs no complications)	1.2 (0.81–1.7)	.38
Total major complications (vs no complications)	0.71 (0.26–1.9)	.5
Previous device interventions		
Per additional previous intervention	0.96 (0.89–1)	.3
History of TLE	0.8 (0.58–1.1)	.18

Reference group is “yes vs no” unless stated otherwise. CI = confidence interval; HR = hazard ratio; TLE = transvenous lead extraction; other abbreviations as in Table 1.
