# Supplementary material for: Long-term survival following transvenous lead extraction: Importance of indication and comorbidities
Source: Heart Rhythm. Author manuscript; Available in PMC 2024 Dec 9. (PMC7617167; doi:10.1016/j.hrthm.2021.05.007)
Supplement: Supplementary material [file EMS198160-supplement-Supplementary_material.docx]

**Table S1**

|  | **Infection** | | | | **Non-Infection** | | | |  |  |
| --- | --- | --- | --- | --- | --- | --- | --- | --- | --- | --- |
|  | **Total** | **Alive** | **Dead** | **p-value*** | **Total** | **Alive** | **Dead** | **p-value**† | **p-value**‡ | **p-value**§ |
| Total Number of Patients | 632 | 388 | 244 |  | 519 | 371 | 148 |  | - |  |
| Follow up time in months (median [IQR]) | 65.15 [22.05, 129.17] | 74.80 [25.20, 151.75] | 53.80 [16.00, 103.90] | <0.001 | 59.00 [32.00, 92.00] | 66.00 [38.00, 95.00] | 42.50 [22.75, 70.25] | <0.001 | 0.98 | 0.785 |
| **Gender** |  |  |  |  |  |  |  |  |  |  |
| Male (%) | 487 (77.1) | 287 (74.0) | 200 (82.0) | 0.026 | 347 (66.9) | 235 (63.3) | 112 (75.7) | 0.01 | 0.171 | <0.001 |
| Explant Age in Years (mean (SD)) | 67.59 (13.59) | 63.58 (13.91) | 73.98 (10.24) | <0.001 | 61.47 (15.35) | 58.18 (15.25) | 69.74 (12.19) | <0.001 | <0.001 | <0.001 |
| >60 years old | 781 (67.9) |  |  |  |  |  |  |  |  |  |
| **Dwell Time** |  |  |  |  |  |  |  |  |  |  |
| Dwell Time in Months (median [IQR]) | 65.15 [22.05, 129.17] | 74.80 [25.20, 151.75] | 53.80 [16.00, 103.90] | <0.001 | 61.00 [15.95, 107.90] | 66.50 [17.25, 113.90] | 52.75 [14.52, 81.65] | 0.05 | 0.401 | 0.022 |
| Dwell Time in Years (median [IQR]) | 5.50 [1.90, 10.80] | 6.20 [2.10, 12.67] | 4.60 [1.40, 8.80] | <0.001 | 5.40 [1.70, 9.20] | 5.90 [1.80, 9.62] | 4.60 [1.30, 6.80] | 0.026 | 0.51 | 0.097 |
| **Type of infection** |  |  |  |  |  |  |  |  |  |  |
| Local Infection | 423 (67.0) | 256 (66.1) | 167 (68.4) | 0.61 | - | - | - | - | - |  |
| Systemic Infection | 209 (33.1) | 132 (34.0) | 77 (31.6) | 0.58 | - | - | - | - | - |  |
| **Non-Infective Indication** |  |  |  |  |  |  |  |  |  |  |
| Lead Dysfunction (%) | - | - | - | - | 342 (65.9) | 240 (64.7) | 102 (68.9) | 0.415 | - |  |
| Functional Lead (%) | - | - | - | - | 31 (6.0) | 24 (6.5) | 7 (4.8) | 0.589 | - |  |
| Lead Complication (%) | - | - | - | - | 69 (13.3) | 43 (11.6) | 26 (17.6) | 0.095 | - |  |
| Lead Access (%) | - | - | - | - | 47 (9.1) | 32 (8.6) | 15 (10.2) | 0.7 | - |  |
| Lead Pain (%) | - | - | - | - | 10 (1.9) | 10 (2.7) | 0 (0.0) | 0.097 | - |  |
| Other indication (%) | - | - | - | - | 97 (18.7) | 66 (17.8) | 31 (20.9) | 0.479 | - |  |
| **Lead Type and number** |  |  |  |  |  |  |  |  |  |  |
| Single Coil Defibrillator Leads (%) |  |  |  | 0.445 |  |  |  | 0.328 | 0.037 | 0.005 |
| 1 | 104 (16.5) | 61 (15.7) | 43 (17.6) |  | 124 (23.9) | 84 (22.6) | 40 (27.0) |  |  |  |
| 2 | 2 (0.3) | 2 (0.5) | 0 (0.0) |  | 3 (0.6) | 3 (0.8) | 0 (0.0) |  |  |  |
| Dual Coil Defibrillator Leads (%) |  |  |  | 0.252 |  |  |  | 0.776 | 0.347 | 0.006 |
| 1 | 109 (17.2) | 60 (15.5) | 49 (20.1) |  | 121 (23.3) | 85 (22.9) | 36 (24.3) |  |  |  |
| 2 | 8 (1.3) | 6 (1.5) | 2 (0.8) |  | 1 (0.2) | 1 (0.3) | 0 (0.0) |  |  |  |
| No. of LV leads (%) |  |  |  | 0.148 |  |  |  | <0.001 | 0.451 | <0.001 |
| 1 | 151 (23.9) | 82 (21.1) | 69 (28.3) |  | 74 (14.3) | 36 (9.7) | 38 (25.7) |  |  |  |
| 2 | 5 (0.8) | 3 (0.8) | 2 (0.8) |  | 4 (0.8) | 4 (1.1) | 0 (0.0) |  |  |  |
| 3 | 2 (0.3) | 2 (0.5) | 0 (0.0) |  | - | - | - |  |  |  |
| Total Leads Extracted (%)* |  |  |  | 0.936 |  |  |  | 0.003 | <0.001 | <0.001 |
| 1 | 105 (16.6) | 63 (16.2) | 42 (17.2) |  | 224 (43.2) | 163 (43.9) | 61 (41.2) |  |  |  |
| 2 | 302 (47.8) | 191 (49.2) | 111 (45.5) |  | 203 (39.1) | 154 (41.5) | 49 (33.1) |  |  |  |
| 3 | 154 (24.4) | 90 (23.2) | 64 (26.2) |  | 68 (13.1) | 44 (11.9) | 24 (16.2) |  |  |  |
| 4 | 54 (8.5) | 34 (8.8) | 20 (8.2) |  | 19 (3.7) | 7 (1.9) | 12 (8.1) |  |  |  |
| 5 | 14 (2.2) | 8 (2.1) | 6 (2.5) |  | 3 (0.6) | 1 (0.3) | 2 (1.4) |  |  |  |
| 6 | 3 (0.5) | 2 (0.5) | 1 (0.4) |  | 2 (0.4) | 2 (0.5) | 0 (0.0) |  |  |  |
| 7 |  |  |  |  | - | - | - |  |  |  |
| **Indication for CIED** |  |  |  |  |  |  |  |  |  |  |
| Primary Prevention | 50 (7.9) | 36 (9.3) | 14 (5.7) | 0.146 | 63 (12.1) | 48 (12.9) | 15 (10.1) | 0.463 | 0.158 | 0.022 |
| Secondary Prevention | 106 (16.8) | 68 (17.5) | 38 (15.6) | 0.596 | 127 (24.5) | 100 (27.0) | 27 (18.2) | 0.049 | 0.583 | 0.002 |
| Any Pacing Indication | 350 (55.4) | 223 (57.5) | 127 (52.0) | 0.21 | 210 (40.5) | 160 (43.1) | 50 (33.8) | 0.063 | 0.001 | <0.001 |
| Any HF indication | 143 (22.6) | 75 (19.3) | 68 (27.9) | 0.016 | 125 (24.1) | 67 (18.1) | 58 (39.2) | <0.001 | 0.027 | 0.608 |
| **Echocardiographic Findings** |  |  |  |  |  |  |  |  |  |  |
| LVEF (mean (SD)) | 46.44 (13.49) | 48.45 (12.89) | 43.12 (13.83) | <0.001 | 44.02 (14.57) | 47.62 (13.35) | 35.55 (13.84) | <0.001 | <0.001 | 0.007 |
| Presence of Vegetation | 85 (13.4) | 62 (16.0) | 23 (9.4) | 0.026 | - | - | - | - | - |  |
| Vegetation >10mm | 36 (5.7) | 30 (7.7) | 6 (2.5) | 0.009 | - | - | - | - | - |  |
| Pacing Lead Vegetation | 66 (10.4) | 49 (12.6) | 17 (7.0) | 0.033 | - | - | - | - | - |  |
| **Co-Morbidities** |  |  |  |  |  |  |  |  |  |  |
| Ischaemic Heart Disease | 243 (40.2) | 123 (32.9) | 120 (51.9) | <0.001 | 182 (36.0) | 100 (27.7) | 82 (56.6) | <0.001 | 0.444 | 0.17 |
| CABG | 86 (14.2) | 38 (10.1) | 48 (21.0) | <0.001 | 57 (11.3) | 27 (7.5) | 30 (20.7) | <0.001 | 1 | 0.167 |
| Valve Disease | 72 (11.9) | 39 (10.4) | 33 (14.3) | 0.189 | 39 (7.7) | 19 (5.3) | 20 (13.8) | 0.002 | 1 | 0.026 |
| Heart Failure | 217 (35.8) | 114 (30.4) | 103 (44.6) | 0.001 | 201 (39.6) | 112 (30.9) | 89 (61.4) | <0.001 | 0.002 | 0.21 |
| Diabetes Mellitus | 112 (18.7) | 67 (18.0) | 45 (20.0) | 0.61 | 62 (12.3) | 38 (10.5) | 24 (16.7) | 0.08 | 0.507 | 0.004 |
| Hypertension | 250 (41.8) | 140 (37.5) | 110 (48.9) | 0.008 | 184 (36.5) | 119 (33.0) | 65 (45.5) | 0.012 | 0.592 | 0.083 |
| Peripheral Vascular Disease | 27 (4.5) | 14 (3.8) | 13 (5.8) | 0.341 | 16 (3.2) | 5 (1.4) | 11 (7.6) | 0.001 | 0.624 | 0.32 |
| Stroke | 57 (9.5) | 33 (8.8) | 24 (10.6) | 0.567 | 30 (5.9) | 16 (4.4) | 14 (9.7) | 0.038 | 0.919 | 0.036 |
| Chronic Respiratory Disease | 72 (12.0) | 39 (10.5) | 33 (14.7) | 0.161 | 75 (14.9) | 50 (13.9) | 25 (17.2) | 0.412 | 0.604 | 0.201 |
| Chronic Kidney Disease | 129 (21.1) | 54 (14.3) | 75 (31.9) | <0.001 | 79 (15.6) | 40 (11.0) | 39 (27.1) | <0.001 | 0.379 | 0.022 |
| Total Number of co-morbidities (%)* |  |  |  | <0.001 |  |  |  | <0.001 | 0.362 | 0.478 |
| 0 | 173 (27.4) | 130 (33.5) | 43 (17.6) |  | 153 (29.5) | 138 (37.2) | 15 (10.1) |  |  |  |
| 1 | 108 (17.1) | 74 (19.1) | 34 (13.9) |  | 107 (20.6) | 85 (22.9) | 22 (14.9) |  |  |  |
| 2 | 123 (19.5) | 68 (17.5) | 55 (22.5) |  | 100 (19.3) | 68 (18.3) | 32 (21.6) |  |  |  |
| 3 | 96 (15.2) | 55 (14.2) | 41 (16.8) |  | 72 (13.9) | 39 (10.5) | 33 (22.3) |  |  |  |
| 4 | 69 (10.9) | 33 (8.5) | 36 (14.8) |  | 52 (10.0) | 26 (7.0) | 26 (17.6) |  |  |  |
| 5 | 35 (5.5) | 17 (4.4) | 18 (7.4) |  | 19 (3.7) | 7 (1.9) | 12 (8.1) |  |  |  |
| 6 | 24 (3.8) | 8 (2.1) | 16 (6.6) |  | 13 (2.5) | 7 (1.9) | 6 (4.1) |  |  |  |
| 7 | 4 (0.6) | 3 (0.8) | 1 (0.4) |  | 3 (0.6) | 1 (0.3) | 2 (1.4) |  |  |  |
| **Pre extraction biochemistry** |  |  |  |  |  |  |  |  |  |  |
| Creatinine Level (median [IQR]) | 96.00 [79.00, 121.00] | 89.00 [77.00, 110.00] | 104.50 [87.00, 137.00] | <0.001 | 87.00 [72.00, 111.00] | 82.00 [70.00, 100.75] | 106.00 [85.00, 139.00] | <0.001 | 0.947 | <0.001 |
| eGFR (mean (SD)) | 65.57 (21.36) | 70.75 (18.85) | 57.34 (22.54) | <0.001 | 69.47 (20.95) | 74.15 (18.41) | 57.74 (22.37) | <0.001 | 0.866 | 0.002 |
| Peak CRP (median [IQR]) | 8.00 [3.00, 22.00] | 6.00 [2.00, 19.00] | 10.00 [5.00, 27.75] | 0.004 | 5.00 [1.00, 11.00] | 5.00 [1.00, 8.00] | 6.00 [1.00, 12.00] | 0.195 | <0.001 | <0.001 |
| **Microbiology Results** |  |  |  |  |  |  |  |  |  |  |
| Positive Microbiology | 411 (65.0) | 273 (70.4) | 138 (56.6) | 0.001 | - | - | - | - | - |  |
| Positive Blood Cultures | 136 (21.5) | 100 (25.8) | 36 (14.8) | 0.001 | - | - | - | - | - |  |
| Positive Swab Cultures | 158 (25.0) | 90 (23.2) | 68 (27.9) | 0.22 | - | - | - | - | - |  |
| Positive Lead Cultures | 272 (43.0) | 170 (43.8) | 102 (41.8) | 0.678 | - | - | - | - | - |  |
| **Previous Device Procedures** |  |  |  |  |  |  |  |  |  |  |
| History of Previous Extraction | 67 (10.6) | 41 (10.6) | 26 (10.7) | 1 | 61 (11.8) | 46 (12.4) | 15 (10.1) | 0.567 | 1 | 0.6 |
| **No. of Previous Device Interventions** |  |  |  | 0.083 |  |  |  | 0.313 | <0.001 | <0.001 |
| 0 | 318 (50.3) | 179 (46.1) | 139 (57.0) |  | 156 (30.1) | 111 (30.0) | 45 (30.4) |  |  |  |
| 1 | 145 (22.9) | 94 (24.2) | 51 (20.9) |  | 207 (40.0) | 142 (38.4) | 65 (43.9) |  |  |  |
| 2 | 86 (13.6) | 55 (14.2) | 31 (12.7) |  | 84 (16.2) | 57 (15.4) | 27 (18.2) |  |  |  |
| 3 or more | 83 (13.1) | 60 (15.4) | 46 (18.9) |  | 71 (12.7) | 60 (16.2) | 11 (7.5) |  |  |  |
| **Extraction Tools*** |  |  |  |  |  |  |  |  |  |  |
| Manual Traction Only (%) | 155 (24.5) | 93 (24.0) | 62 (25.4) | 0.753 | 93 (17.9) | 69 (18.6) | 24 (16.2) | 0.609 | 0.581 | 0.734 |
| Non-powered only (%) | 128 (20.3) | 69 (17.8) | 59 (24.2) | 0.065 | 78 (15.0) | 47 (12.7) | 31 (20.9) | 0.025 | 0.539 | 0.026 |
| Powered Only (%) | 62 (9.8) | 38 (9.8) | 24 (9.8) | 1 | 57 (11.0) | 37 (10.0) | 20 (13.5) | 0.313 | 0.258 | 0.212 |
| Powered and Non-Powered (%) | 287 (45.4) | 188 (48.5) | 99 (40.6) | 0.064 | 220 (42.4) | 162 (43.7) | 58 (39.2) | 0.405 | 0.737 | <0.001 |
| **Extraction Approach** |  |  |  |  |  |  |  |  |  |  |
| Inferior Approach (%) | 67 (10.7) | 48 (12.5) | 19 (7.8) | 0.085 | 50 (9.7) | 44 (11.9) | 6 (4.1) | 0.01 | 0.21 | 0.647 |
| Primary Femoral Approach (%) | 11 (1.7) | 7 (1.8) | 4 (1.6) | 1 | 3 (0.6) | 3 (0.8) | 0 (0.0) | 0.647 | 0.295 | 0.127 |
| Secondary Femoral Approach (%) | 60 (9.5) | 45 (11.7) | 15 (6.1) | 0.03 | 49 (9.5) | 43 (11.6) | 6 (4.1) | 0.013 | 0.509 | 1 |
| **Pacing during extraction** |  |  |  |  |  |  |  |  |  |  |
| Temporary Pacing Wire (%) | 200 (31.6) | 126 (32.5) | 74 (30.3) | 0.633 | 68 (13.1) | 50 (13.5) | 18 (12.2) | 0.797 | <0.001 | <0.001 |
| **Procedural Success*** |  |  |  |  |  |  |  |  |  |  |
| Complete Remove | 574 (90.8) | 351 (90.5) | 223 (91.4) | 0.801 | 450 (86.7) | 326 (87.9) | 124 (83.8) | 0.274 | 0.004 | 0.001 |
| Partial Removal | 52 (8.2) | 34 (8.8) | 18 (7.4) | 0.639 | 63 (12.1) | 39 (10.5) | 24 (16.2) | 0.099 | 0.023 | 0.068 |
| Clinical Failure | 6 (0.9) | 3 (0.8) | 3 (1.2) | 0.877 | 6 (1.2) | 6 (1.6) | 0 (0.0) | 0.271 | 0.449 | 0.959 |
| **Complications** |  |  |  |  |  |  |  |  |  |  |
| All Minor Complications | 56 (8.9) | 36 (9.3) | 20 (8.2) | 0.747 | 43 (8.3) | 34 (9.2) | 9 (6.1) | 0.33 | 0.564 | 0.81 |
| Total Major Complications | 14 (2.2) | 10 (2.6) | 4 (1.6) | 0.615 | 8 (1.5) | 8 (2.2) | 0 (0.0) | 0.16 | 0.295 | 0.539 |

†**- the p value is a when comparing the alive and dead groups of each cohort**

‡ **- the p value is when comparing the dead groups of the infection and non-infection cohorts**

§ **- the p value is when comparing the total (i.e., dead and alive) infection and non-infection cohorts**

*** - these categories are mutually exclusive (i.e. the totals of these sub-categories represent 100% of the total in each subgroup)**

**Table S2**

|  | **Infection Group** | | **Non-Infection Group** | |
| --- | --- | --- | --- | --- |
|  | **HR (CI)** | **p-value** | **HR (CI)** | **p-value** |
|  |  |  |  |  |
| Explant Age in Years (per year) | 1.1 (1.1-1.1) | <0.001 | 1.1 (1-1.1) | <0.001 |
| Explant Age>60 years (yes vs no) | 4.5 (2.9-6.8) | <0.002 | 2.9 (1.98-4.36) | <0.001 |
| Gender (male vs female) | 1.6 (1.1-2.2) | 0.0087 | 1.5 (1-2.2) | 0.031 |
| Dwell Time in Years (per additional year) | 0.96 (0.94-0.98) | <0.001 | 0.98 (0.96-1) | 0.23 |
| **Lead Type** |  |  |  |  |
| Dual Coil Defibrillator Leads (vs Single Coil) | 1.2 (0.82-1.7) | 0.38 | 1.1 (0.67-1.6) | 0.84 |
| No. of LV leads (per additional LV lead) | 1.6 (1.3-2.1) | <0.001 | 2.2 (1.5-3.1) | <0.001 |
| Total Leads Extracted (per additional lead) | 1.2 (1-1.3) | 0.0082 | 1.3 (1.1-1.5) | 0.003 |
| **Indication for CIED** |  |  |  |  |
| Primary Prevention (vs Secondary Prevention) | 0.95 (0.52-1.7) | 0.85 | 1.08 (0.63-1.8) | 0.79 |
| Any Pacing Indication (yes vs no) | 0.71 (0.55-0.91) | 0.0071 | 0.7 (0.49-0.98) | 0.038 |
| Any HF indication (yes vs no) | 2 (1.5-2.7) | <0.001 | 2.8 (2-3.9) | <0.001 |
| **Echocardiographic Findings** |  |  |  |  |
| LVEF (per % increase) | 0.98 (0.97-0.99) | <0.001 | 0.95 (0.94-0.97) | <0.001 |
| Presence of Vegetation (yes vs no) | 0.82 (0.54-1.3) | 0.37 | - | - |
| Vegetation >10mm (yes vs no) | 0.49 (0.22-1.1) | 0.085 | - | - |
| Pacing Lead Vegetation (yes vs no) | 0.75 (0.46-1.2) | 0.25 | - | - |
| **Microbiology Results** |  |  |  |  |
| Positive Microbiology (yes vs no) | 0.83 (0.65-1.1) | 0.16 | - | - |
| Positive Blood Cultures (yes vs no) | 1.2 (0.87-1.8) | 0.23 | - | - |
| Positive Swab Cultures (yes vs no) | 1.2 (0.89-1.6) | 0.24 | - | - |
| **Indication for Extraction** |  |  |  |  |
| Any Infective Indication (yes vs no) | - | - | - | - |
| Local Infection (yes vs no) | 1.1 (0.81-1.4) | 0.65 | - | - |
| Systemic Infection (yes vs no) | 0.94 (0.71-1.2) | 0.64 | - | - |
| Non-Infective Indication |  |  |  |  |
| Lead Dysfunction (yes vs no) | - | - | 1.1 (0.81-1.6) | 0.44 |
| Functional Lead (yes vs no) | - | - | 0.61 (0.29-1.3) | 0.2 |
| Lead Complication (yes vs no) | - | - | 1.3 (0.83-1.9) | 0.26 |
| Lead Access (yes vs no) | - | - | 1.4 (0.81-2.3) | 0.24 |
| Lead Pain (yes vs no) | - | - | 1.1e-07 (0-Inf) | 0.99 |
| Other indication (yes vs no) | - | - | 1.2 (0.79-1.8) | 0.41 |
| **Co-Morbidities** |  |  |  |  |
| Ischaemic Heart Disease (yes vs no) | 1.9 (1.4-2.4) | <0.001 | 2.7 (2-3.8) | <0.001 |
| CABG (yes vs no) | 1.6 (1.2-2.2) | 0.0046 | 2.5 (1.6-3.7) | <0.001 |
| Valve Disease (yes vs no) | 1.6 (1.1-2.3) | 0.016 | 2.3 (1.4-3.7) | <0.001 |
| Heart Failure (yes vs no) | 2.4 (1.8-3.1) | <0.001 | 3.3 (2.4-4.7) | <0.001 |
| Diabetes Mellitus (yes vs no) | 1.5 (1.1-2.1) | 0.012 | 1.8 (1.2-2.8) | 0.0094 |
| Hypertension (yes vs no) | 1.8 (1.4-2.4) | <0.001 | 1.7 (1.3-2.4) | 0.001 |
| Peripheral Vascular Disease (yes vs no) | 1.6 (0.91-2.8) | 0.1 | 4.1 (2.2-7.7) | <0.001 |
| Stroke (yes vs no) | 1.6 (1-2.4) | 0.03 | 2.4 (1.4-4.3) | 0.0017 |
| Chronic Respiratory Disease (yes vs no) | 2 (1.4-3) | <0.001 | 1.4 (0.94-2.2) | 0.095 |
| Chronic Kidney Disease (yes vs no) | 2.9 (2.2-3.9) | <0.001 | 3.5 (2.4-5.1) | <0.001 |
| Total Number of co-morbidities (yes vs no) | 1.3 (1.2-1.4) | <0.001 | 1.5 (1.4-1.7) | <0.001 |
| **Pre extraction biochemistry** |  |  |  |  |
| Creatinine Level (per 10mg/dL increase) | 1.08 (1.1-1.1) | <0.001 | 1.09 (1.07-1.11) | <0.001 |
| eGFR (per increase in ml/min/1.73m2) | 0.98 (0.97-0.98) | <0.001 | 0.98 (0.97-0.98) | <0.001 |
| Peak CRP (per increase in mg/dL) | 1 (1.002-1.007) | <0.001 | 1 (0.996-1.008) | 0.53 |
| **Extraction Technique** |  |  |  |  |
| Manual Traction Only (yes vs no) | 1.1 (0.8-1.5) | 0.55 | 0.88 (0.57-1.4) | 0.57 |
| Non-powered only (vs powered) | 1.2 (0.72-1.9) | 0.56 | 1.01 (0.57-1.8) | 0.98 |
| Powered and Non-Powered (vs manual traction only) | 0.9 (0.54-1.4) | 0.56 | 0.92 (0.61-1.4) | 0.71 |
| Inferior Approach (vs superior approach) | 1.1 (0.66-1.7) | 0.82 | 0.62 (0.27-1.4) | 0.26 |
| Secondary Femoral Approach (vs primary femoral approach) | 1.1 (0.34-3.3 | 0.91 | 2.6e-04 (0-Inf) | 1 |
| Surgical Extraction (yes vs no) | 0.6 (0.25-1.5) | 0.28 | 1.1e-07 (0-Inf) | 0.99 |
| **Pacing during extraction** |  |  |  |  |
| Temporary Pacing Wire (yes vs no) | 1.1 (0.81-1.4) | 0.68 | 1 (0.61-1.6) | 1 |
| External Pacing (yes vs no) | 1.4 (1-2) | 0.049 | 1 (0.41-2.4) | 1 |
| **Procedural Success** |  |  |  |  |
| Complete Remove (yes vs no) | 1.1 (0.7-1.7) | 0.74 | 0.93 (0.62-1.4) | 0.72 |
| Partial Removal (vs complete removal) | 0.94 (0.6-1.5) | 0.8 | 1.28 (0.83-2) | 0.27 |
| Clinical Failure (yes vs no) | 0.86 (0.27-2.7) | 0.79 | 1.1e-07 (0-Inf) | 0.99 |
| All Minor Complications (vs no complications) | 1.3 (0.82-2.1) | 0.26 | 0.98 (0.5-1.9) | 0.96 |
| Total Major Complications (vs no complications) | 0.94 (0.35-2.5) | 0.9 | 3e-07 (0-Inf) | 0.99 |
| **Previous Device interventions** |  |  |  |  |
| No. of Previous Device Interventions (per additional intervention) | 1 (0.91-1.1) | 0.99 | 0.93 (0.82-1.1) | 0.26 |
| History of Previous TLE (yes vs no) | 1.1 (0.7-1.6) | 0.8 | 0.72 (0.42-1.2) | 0.24 |
| Number of Previous TLEs (per additional TLE procedure) | 1.1 (0.86-1.4) | 0.45 | 0.75 (0.51-1.1) | 0.13 |

Univariate Cox regression model to predict long term mortality after TLE in the infection and non-infection subgroups

**Table S3**

Multivariate Cox regression model to predict mortality after transvenous lead extraction (TLE) in the infection (model significance, p<0.001) and non-infection group (model significance, p<0.001).

|  | **Infection** | | **Non-Infection Group** | |
| --- | --- | --- | --- | --- |
|  | **HR (CI)** | **p-value** | **HR (CI)** | **p-value** |
| **Male Gender** | 0.98 (0.47-2.06) | 0.961 | 0.79 (0.51-1.23) | 0.303 |
| **Number of LV leads Extracted** | 1.17 (0.58-2.34) | 0.656 | 0.84 (0.46-1.55) | 0.582 |
| **Any Pacing Indication** | 1.02 (0.70-1.47) | 0.927 | 0.94 (0.58-1.5) | 0.786 |
| **LVEF (per % increase)** | 0.98 (0.97-1.00) | 0.016 | 0.97 (0.95-0.99) | <0.001 |
| **Peak CRP (per mg/dL)** | 1.01 (1.00-1.01) | <0.001 | - | - |
| **Age >75 years** | 3.20 (2.33-4.40) | <0.001 | 3.04 (2.03-4.56) | <0.001 |
| **Number of leads extracted** | 1.18 (0..98-1.42) | 0.084 | 1.04 (0.83-1.31) | 0.739 |
| **Number of co-morbidities** | 1.17 (1.06-1.28) | 0.001 | 1.2 (1.06-1.37) | 0.005 |
| **Heart Failure Indication** | 0.98 (0.47-2.06) | 0.961 | 1.08 (0.63-1.84) | 0.785 |
| **Dwell time (years)** | 0.98 (0.96-1.01) | 0.191 | 0.98 (0.95-1.01) | 0.167 |
| **eGFR<60 ml/min/1.73m^2^** | 1.57 (1.13-2.18) | 0.007 | 1.81 (1.21-2.72) | 0.004 |

**Figure S1**

**
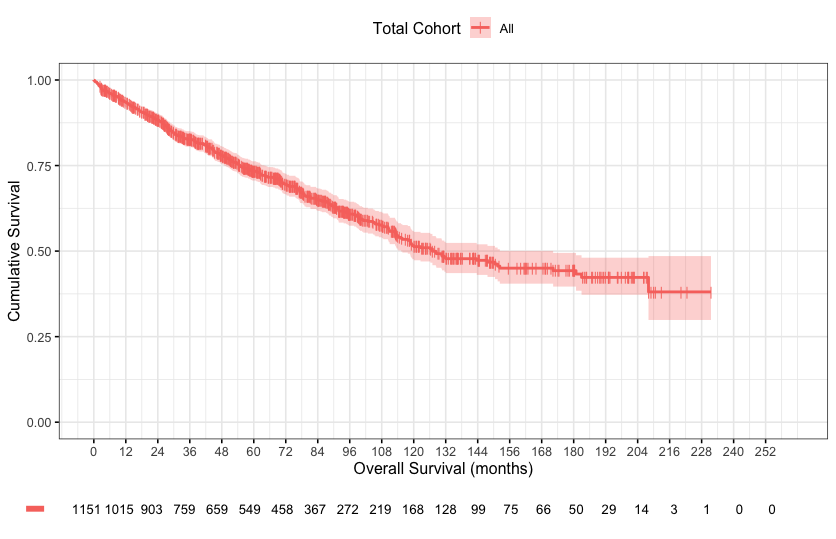
**

Kaplan-Meier survival probability of the Total Cohort

**Figure S2**

**
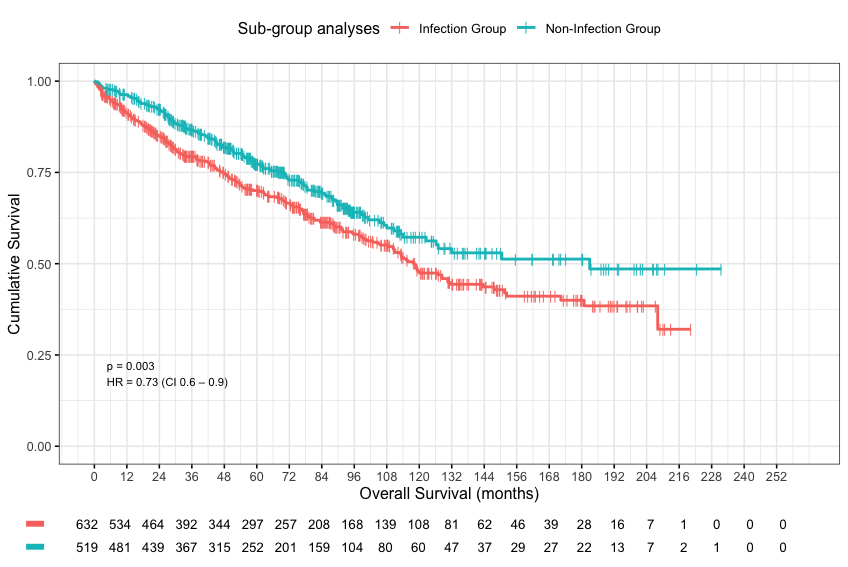
**

Kaplan-Meier survival probability of the infection and non-infection cohorts

**Figure S3**

**
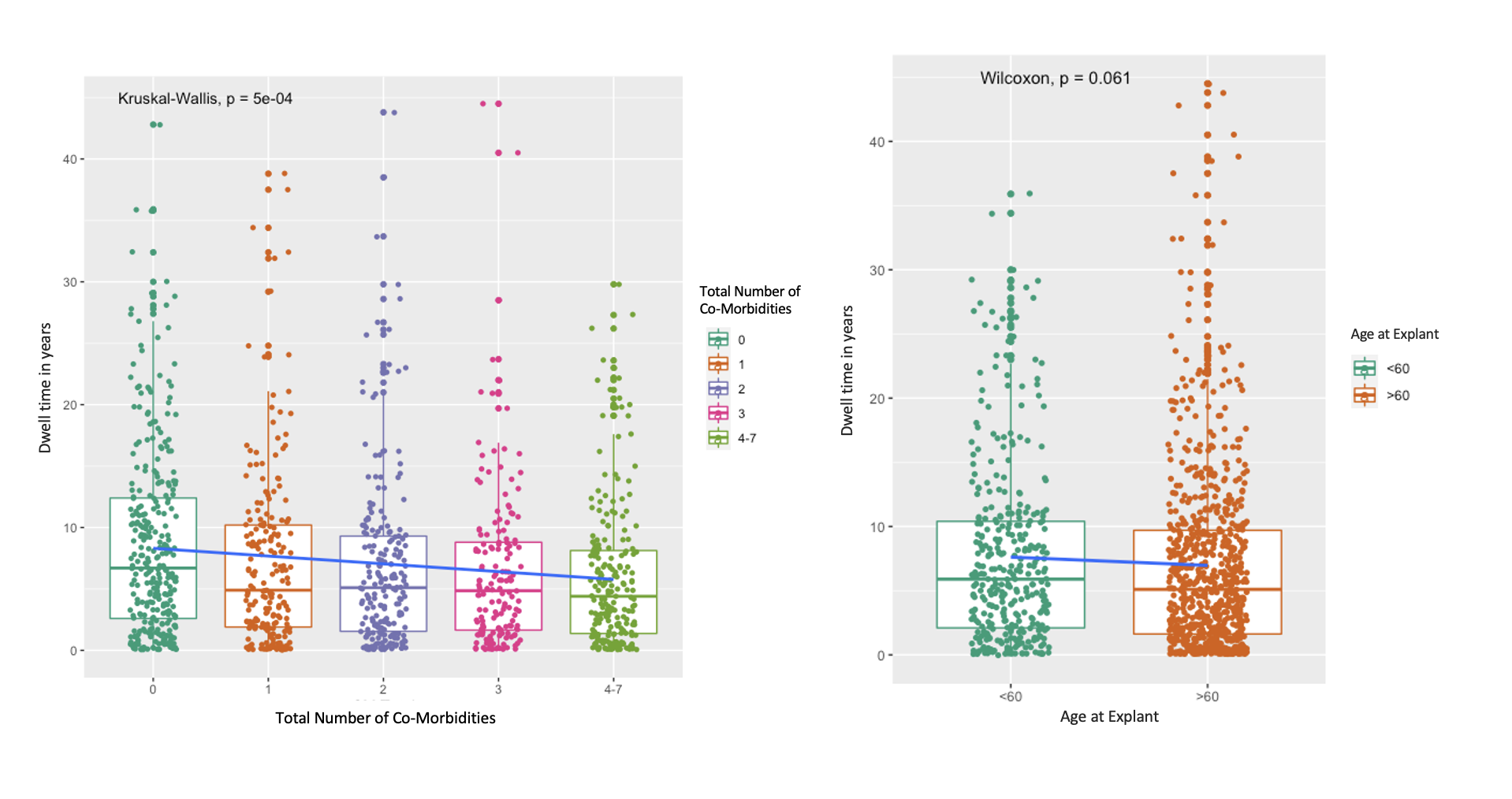
**

Box plot demonstrating relationship between Co-Morbidity burden and age categories against lead dwell time

**Figure S4**

**
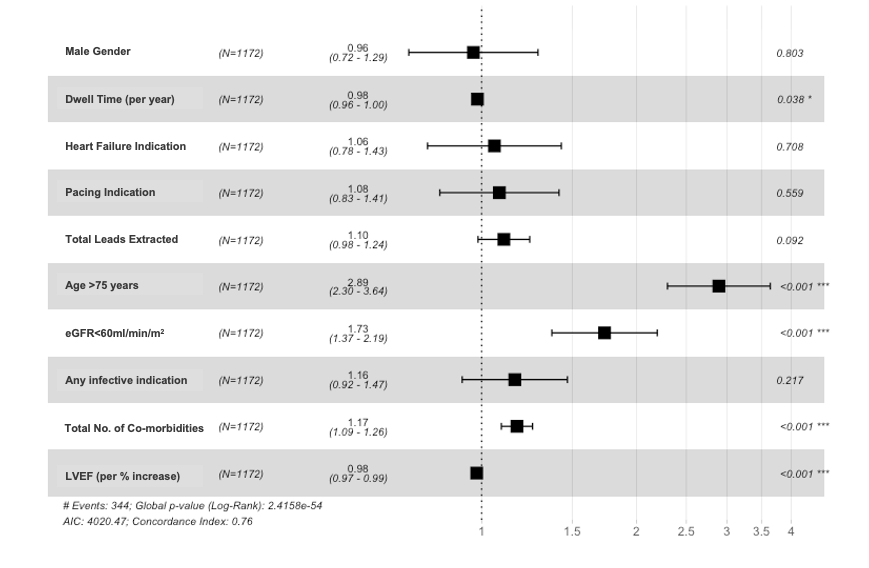
**

Competing multivariable cox proportional hazards regression model (p<0.001) to predict mortality after TLE in the Total Cohort including hospital mortality.
