# Supplementary material for: Long-term survival following transvenous lead extraction: Importance of indication and comorbidities
Source: Heart Rhythm. Author manuscript; Available in PMC 2024 Dec 9. (PMC7617167; doi:10.1016/j.hrthm.2021.05.007)
Supplement: Appendix [file EMS198160-supplement-Appendix.pdf]

Appendix

Supplementary data

Supplementary data associated with this article can be found in the online version at https://doi.org/10.1016/j.hrthm.2021.05.007.
